# 

**Published:** 1920-02

**Authors:** 


### ANNOUNCEMENTS

### French-Belgian Relief Fund

*The Preparedness League* is urging all dentists to send contributions to this cause, as it is a very urgent need, and any money sent now will be more welcome and useful than at any other time. A committee of American dentists in France is co-operating with the League and will see that all contributions are used to the best advantage. All money should be sent to the treasurer of the League, Dr. L. M. Waugh, 576 Fifth Ave., New York City, N. Y.

*Michigan State Dental Society* meets April 12, 13 and 14, in Detroit.

*American Society of Orthodontists* will meet April 5, 6 and 7 at Edgewater Beach Hotel, in Chicago.

*West Virginia State Society* meets in Charleston, April 14-16, 1920.

*Illinois State and Chicago Dental Societies* are planning a joint session in Chicago, March 22-25, at Congress Hotel. Great preparations are being made for a big event.

*Kentucky State Dental Society* meets in Louisville, April 7-9, 1920. An unusually good program is in preparation.

### Annual Convention Texas State Dental Society

The Texas State Dental Society will hold its fortieth annual convention March 8-13, 1920, at Dallas, Texas.

The special feature of this meeting will be post-graduate class courses conducted on the "Oklahoma Plan."

These classes will be conducted by the following well-known men: Dr. Thomas B. Hartzell, Minneapolis, Minn.; Dr. F. Ewing Roach, Chicago, Ill.; Dr. Arthur E. Smith,

Chicago, Ill.; Dr. Dayton D. Campbell, Kansas City, Mo., and Dr. T. W. Maves, Minneapolis, Minn. Other instructors may be employed. J. G. Fife, Secy.
736 Wilson Bldg., Dallas, Texas.

